# Content variations in compositions and volatile component in jujube fruits during the blacking process

**DOI:** 10.1002/fsn3.973

**Published:** 2019-02-21

**Authors:** Xin Sun, Duanyin Gu, Quanbin Fu, Lin Gao, Chuan Shi, Rentang Zhang, Xuguang Qiao

**Affiliations:** ^1^ College of Food Science and Engineering Shandong Agricultural University Tai'an China; ^2^ Tai’ an Academy of Agriculture Sciences Tai'an China

**Keywords:** blacking process, compositions, jujube, volatile component

## Abstract

Dried jujube (*Ziziphus jujuba*) was incubated at high temperature and humidity for 96 hr in blacking process and sampled every 12 hr. Results showed that the saccharose reduced from 195.6 to 3.1 g/kg rapidly in 24 hr. The total acid content was mild with 8.82 g/kg and increased to 23.45 g/kg by 177.21% with thermal processing for 96 hr. The contents of total polyphenols were enhanced during 0–48 hr processing, and the amount of the compound increased with treatment by 50.99%. The total reducing sugar increased 29.79% on 60 hr. cAMP was decreased with aging and ripening by 65.85%. 5‐HMF was keep growing to 3.52 g/kg. The volatile component had great change in black jujube fruits compared to untreated jujubes, especially treated in 12 hr. The results indicated that backing pretreatment can facilitate the generation of functional food materials and support the development of this nutrition product.

## 1 INTRODUCTION

Jujube (*Ziziphus jujuba* Mill.) belongs to the Rhamnaceae family and was originally grown in the mountains, hills, or plains at altitudes below 1,700 m in subtropical and tropical regions of Asia, and it has also been cultivated in Europe and in the Americas in recent years (Gao, Wu, & Wang, 2013). The jujube is cultivated in China on a total of 1.5 million hectares to produce 400,000 tons of fruit, which makes China output account for 98% of the total output worldwide. Jujube fruits have a huge potential for development and economic benefits as part of the economic forest. Jujube fruit is well known for its high nutritional content (Li, Fan, Ding, & Ding, 2007), including common sugars (Chen et al., 2015), acids, and vitamins (Wojdyło, Figiel et al., 2016). Natural bioactive substances that have antioxidant effects, such as triterpenic acid (Gong, Zhao, Liu, Hu, & Gao, 2014; Guo et al., 2015), phenolic compounds (Gao et al., 2012; Wojdyło, Carbonell‐Barrachina, Carbonell‐Barrachina, Legua, & Hernández, 2016), cyclic adenosine monophosphate (cAMP) (Bai et al., 2016; Ji et al., 2017), and flavonoids, (Chen et al., 2013) have also been found in recent studies (Gao et al., 2011).

Jujube fruits have been recognized as a nutritious food and have important uses in the diet and in traditional medicine in China (Wojdyło, Figiel et al., 2016). Jujube as a raw material is used in traditional products, such as mud, candied dates, and moon cake. In recent years, new products have also been developed and applied, such as drinks like milk and wine. However, jujube fruits are not applicable for children and elderly men, especially those with high blood sugar (Wang, Cheng, Cao, & Jiang, 2013).

With similar processing as black garlic, black jujube is a processed jujube product that is prepared via heat treatment of the raw jujube at high temperature and in a high humidity environment for natural aging for a certain time after the finished product. When jujube undergoes heat treatment, various physicochemical changes occur in the color, aroma, flavor, and nutrient content. Heat treatment, in particular, brings about nonenzymatic browning reactions, such as the caramelization reaction, Maillard reaction, and other chemical oxidation reactions. Nonenzymatic browning reactions are associated with the formation of strong antioxidant compounds because jujube contains sugar and amino acids (Kim, Kim, Kim, Park, & Lee, 2013). Black jujubes enhance the antioxidant activity via DPPH radical scavenging, which occurs for a long time during aging. (Park et al., 2012). The sugar content is reduced for the specific population in the black jujube during the aging process. However, information on the change in biological activities and chemical constituents is lacking in the literature. In this study, we monitored the changes in sugar, total acid, pentahydroxymethyl furfural (5′‐HMF), total phenols, cAMP, and volatile components during the process of jujube aging to support the development of low‐sugar and nutrition products.

## 2 MATERIALS AND METHODS

### 2.1 Reagents and materials

We obtained 3′,5′‐cyclic AMP and 5′‐HMF from Ziqibio Co., Ltd. (Shanghai, China), fructose, glucose, and sucrose were purchased from Shanghai Yuanye Bio‐Technology Co., Ltd. (Shanghai, China). Other reagents were of analytical grade. Acetonitrile and methanol were both of HPLC grade and purchased from Merck (Darmstadt, Germany). The dry jujubes were varieties of Ningyang jujube provided by the market (Taian, China).

The high‐performance liquid chromatography (HPLC) was performed on a LC‐20A system Shimadzu (Kyoto, Japan), equipped with diode array detector (SPD‐20A) and refractive index detector (RID‐10A). Data acquisitions were performed using Lab Solution software. IMS instrument (FlavourSpec^®^) from Gesellschaft für Analytische Sensorsysteme mbH (G.A.S., Dortmund, Germany) equipped with a heated splitless injector and automatic sampler unit (CTC‐PAL, CTC Analytics AG, Zwingen, Switzerland) was utilized for instrumental aroma analysis. The IMS was equipped with a FS‐SE‐54‐CB‐1 column (15 m × 0.53 mm). IMS data were acquired and identified unknown compounds using GCxIMS Library Search software, and then analyzed by Laboratory Analytical Viewer supplied by G.A.S.

### 2.2 Sample preparation

The treatment of jujube refers to the production method of black garlic. The dry jujubes were cleaned and soaked in water for one hour and sealed with water (600 g/400 ml); then, the entire jujube was placed in a constant temperature and humidity incubator. The jujube was aged and fermented in an 80% humidity environment at 75°C for 96 hr. 500 g jujubes were taken out every 12 hr and then homogenized after removing the nucleus. All the samples mentioned below were obtained from the homogenized jujubes.

### 2.3 The measurement method of color difference

Taking the raw jujube as a comparison, the color difference was measured by the color difference meter for the jujube samples of each period. The color difference was represented by the values of ΔE*ab. L* represents brightness, a* and b* represent chromaticity. *L*1*, *a*1*, and *b*1* were the measured values of untreated jujubes. *L*2*, *a*2*, and *b*2* were the measured values of treated jujubes of each period.

$$<![CDATA\left[\Delta E*ab\;=\;{\left[{\left(\Delta L^{*}\right)}^{2}+\;{\left(\Delta a^{*}\right)}^{2}+\;{\left(\Delta b^{*}\right)}^{2}\right]}^{1/2}.\right]]>$$

$$<![CDATA\left[\Delta a^{*}=a1^{*}-a2^{*};\Delta b^{*}=b1^{*}-b2^{*};\Delta L^{*}=L1^{*}-L2^{*}.\right]]>$$

### 2.4 Determination of total acid content

The total acid content was analyzed using the national standard of GB/T 12456‐2008. The samples (20 g) were homogenized with 80°C boiled water and placed in a boiling water bath for 30 min (shaken 2–3 times). The volume was adjusted to 250 ml with water after it was cooled down. The supernatant was filtered, added 50 ml of water, and then titrated with 0.1 M of sodium hydroxide solution to a pH of 8.3 in a magnetic stirrer.

### 2.5 Analysis of sugar

The measurement method for fructose, glucose, and sucrose was according to the China National Standard protocol GB 5009.8‐2016. Two grams of homogenized samples were weighed in a beaker with 40 mL of water and allowed to mix in a magnetic stirrer for 10 min. 5 mL of zinc acetate (219 g/L) and 5 mL of potassium ferrocyanide (106 g/L) was added. Then the mixed sample was transferred into bottle and injected water to 100ml. The mixtures were extracted via ultrasonic for 30 min. Finally, the supernatant was reconstituted and transferred into vials after filtering through a 0.45‐μm nylon filter. The mixtures were extracted via ultrasonic for 30 min. Finally, the supernatant was reconstituted and transferred into vials after filtering through a 0.45‐μm nylon filter.

A sample of 10 µl was injected into an Inertsil NH_2_ column (250 × 4.6 mm, 5 µm; Shimadzu) and detected using a refractive index detector in 40°C. The elution was carried out using acetonitrile and water (70:30, v/v) at a flow rate of 1.0 ml/min.

The determination method for the reducing sugar was completely in reference to the national standard (GB 5009.7‐2016, China).

### 2.6 Determination of the total phenolic content

The total phenolic extracts were analyzed using the Folin−Ciocalteu phenol reagent method, which was modified on the basis of the reported literature (Gao et al., 2012). A one‐gram sample was dissolved in a 50 ml ethanol solution (70%), then socked for 10 min and extracted via ultrasonification for 30 min at 66°C.

A total of 750 µl of the Folin−Ciocalteu reagent and 750 µl of deionized water were added to 200 μl of the supernatant of the sample extracts. The mixture was added to 1.5 ml of sodium carbonate (10%, w/v) and brought to a 10 ml volume by deionized water and allowed to stand for 10 min at 75°C before measured on a spectrophotometer at an absorbance of 760 nm. A mixture of Folin−Ciocalteu phenol reagents and deionized water was used as the blank.

### 2.7 Analysis of cAMP

The cAMP content was determined based on an assay method (Kou et al., 2015) with some modifications. The samples (2 g) were extracted for 30 min with 100 ml of boiled and distilled water using an ultrasonic washing unit and then centrifuged for 5 min at 1,509.3 *g*; the supernatant was filtered through a 0.45‐μm syringe filter.

The compounds were eluted in an Inertsil ODS‐3 column (250 × 4.6 mm; 5 μm) at a flow rate of 1 ml/min with a mobile phase of a methanol–potassium dihydrogen phosphate solution (0.05 M) mixture (10:90, v/v). The flow rate was 1 ml/min, and the detection temperature was 30°C. The detection wavelength was 254 nm (5‐nm bandwidth), and the injection volume was 10 µl.

### 2.8 5‐HMF analysis

The 5 g samples were homogenized with 10.0 ml of methanol; then, the mixture was stirred with 30.0 ml of water with mixing using a magnetic stirrer for 10 min. The phase was transferred into a 50 ml bottle and fixed with water and extracted via ultrasonic 30 min after it was fully mixed. The supernatant was filtered through a 0.45‐μm syringe filter prior to HPLC.

A total of 10 μl were injected onto the HPLC column. The analytes were separated in the IntertSustain C18 column (250 × 4.6 mm, 5 μm; Shimadzu) at 35°C. The mobile phase was a methanol–water mixture (2:98, v/v). The absorbance wavelength for determination was 282 nm.

### 2.9 Detection of volatile components

Nitrogen of 99.99% purity was used as the drift and carrier gas. Drift gas flow was set at a constant flow of 150 ml/min. Initial carrier gas flow of 2 ml/min, holding 2 min, increased to 100 ml/min at 20 min. The samples were incubated in headspace volume at 40°C lasting 10 min. 1 g jujube sample was transferred into a 20 ml headspace vial, and then incubated in headspace volume at 40 °C for 25 min. 500 μl headspace was injected by a syringe temperature of 45°C into the heated injector in splitless mode. The analytes were separated at 40 °C column and then ionized in the IMS ionization chamber of 45°C.

**Figure 1:**
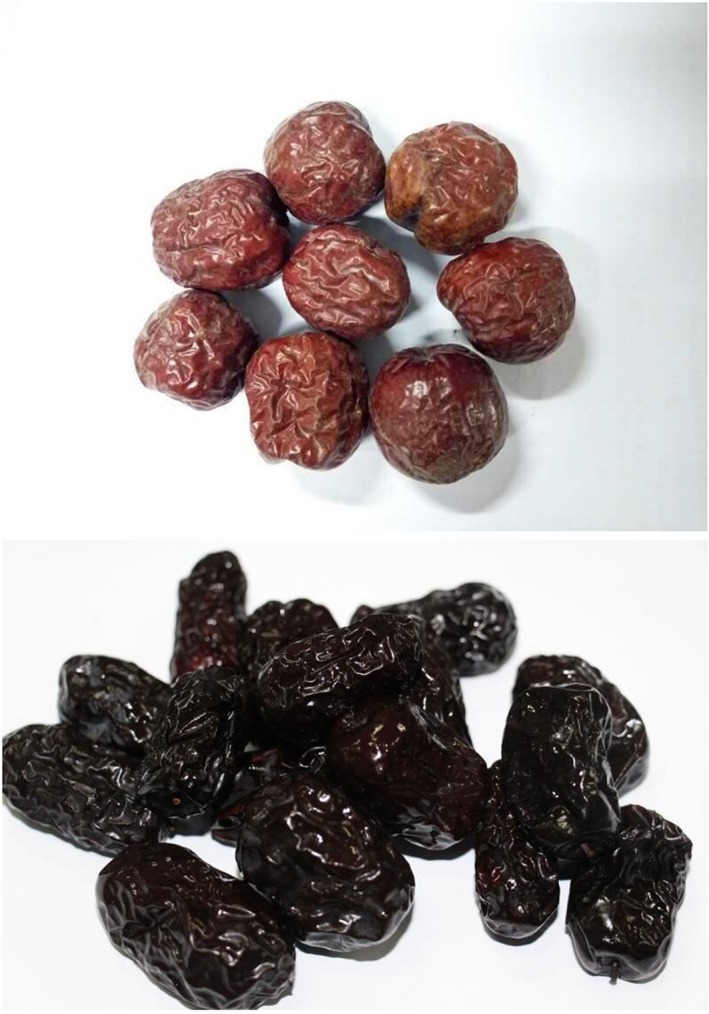
Comparison on the color of raw and black jujubes (96 hr)

A GC–IMS analysis results in a blue picture as shown in Figure 1. It is a two‐dimensional map in which X axis represents the drift time and Y axis represents the retention time. The red vertical line on the left side is the reactive ion (RIP) in the blue background. Each point on entire spectrum represents a volatile organic compound. The color represents the normalized concentration of the substance, white indicates less concentration, and red indicates greater concentration. The darker the color, the greater the concentration. Qualitative data were obtained through NIST database of retention index and IMS migration time database based on GC×IMS Library Search from G.A.S. IMS migration time database was based on GC×IMS Library Search software. Forty‐three qualitative compounds are marked in Figure 1. The information of CAS number, formula, molecular weight (MW), retention index (RI), retention time (RT), migration time (MT) were showed in Table 1.

**Table 1:** The information of 43 qualitative substances

	Compound	CAS#	Formula	MW	RI	RT [s]	DT [RIPrel]
1	Maltol	C118718	C_6_H_6_O_3_	126.1	1,093.4	793.841	1.2103
2	Furaneol	C3658773	C_6_H_8_O_3_	128.1	1,071.9	722.677	1.2019
3	Ethyl hexanoate	C123660	C_8_H_16_O_2_	144.2	1,008.8	548.599	1.8025
4	Ethyl hexanoate	C123660	C_8_H_16_O_2_	144.2	1,008.4	547.504	1.3475
5	1‐Octen‐3‐ol	C3391864	C_8_H_16_O	128.2	986.6	501.521	1.1585
6	Benzaldehyde	C100527	C_7_H_6_O	106.1	954.2	445.685	1.4665
7	Benzaldehyde	C100527	C_7_H_6_O	106.1	954.9	446.779	1.1515
8	2‐Furanmethanol, 5‐methyl‐	C3857258	C_6_H_8_O_2_	112.1	956.9	450.064	1.5687
9	Methyl hexanoate	C106707	C_7_H_14_O_2_	130.2	928	405.176	1.6835
10	Methyl hexanoate	C106707	C_7_H_14_O_2_	130.2	928.7	406.271	1.2901
11	Gamma‐Butyrolactone	C96480	C_4_H_6_O_2_	86.1	908.3	377.259	1.0852
12	Gamma‐Butyrolactone	C96480	C_4_H_6_O_2_	86.1	907.1	375.603	1.3025
13	Ethyl pentanoate	C539822	C_7_H_14_O_2_	130.2	905.5	373.395	1.2752
14	Ethyl pentanoate	C539822	C_7_H_14_O_2_	130.2	903.9	371.186	1.6824
15	Heptanal	C111717	C_7_H_14_O	114.2	901.8	368.426	1.6967
16	Heptanal	C111717	C_7_H_14_O	114.2	901.4	367.874	1.3584
17	2,5‐dimethylpyrazine	C123320	C_6_H_8_N_2_	108.1	905.5	373.395	1.5028
18	2‐Heptanone	C110430	C_7_H_14_O	114.2	893	356.833	1.6316
19	2‐Heptanone	C110430	C_7_H_14_O	114.2	893	356.833	1.2635
20	Ethyl 3‐methylbutanoate	C108645	C_7_H_14_O_2_	130.2	853	316.533	1.2635
21	Ethyl 3‐methylbutanoate	C108645	C_7_H_14_O_2_	130.2	853	316.533	1.6563
22	2‐Hexen‐1‐ol	C2305217	C_6_H_12_O	100.2	848.3	312.117	1.1828
23	2‐Hexen‐1‐ol	C2305217	C_6_H_12_O	100.2	848.3	312.117	1.5145
24	Butyl acetate	C123864	C_6_H_12_O_2_	116.2	810	278.413	1.2392
25	Butyl acetate	C123864	C_6_H_12_O_2_	116.2	810.8	279.025	1.6177
26	Ethyl butanoate	C105544	C_6_H_12_O_2_	116.2	819.5	286.37	1.556
27	Ethyl butanoate	C105544	C_6_H_12_O_2_	116.2	819.1	286.064	1.2178
28	2‐Hexanol	C626937	C_6_H_14_O	102.2	793.4	264.947	1.5636
29	2‐Hexanol	C626937	C_6_H_14_O	102.2	791.9	263.723	1.2869
30	2‐Hexanone	C591786	C_6_H_12_O	100.2	782.3	256.378	1.4995
31	2‐Hexanone	C591786	C_6_H_12_O	100.2	783.1	256.99	1.1914
32	Methyl 2‐methylbutanoate	C868575	C_6_H_12_O_2_	116.2	771.7	249.033	1.5309
33	3‐methylbutanol	C123513	C_5_H_12_O	88.1	730.8	222.407	1.3322
34	Ethyl propanoate	C105373	C_5_H_10_O_2_	102.1	708.7	209.247	1.4542
35	2‐Ethylfuran	C3208160	C_6_H_8_O	96.1	703.3	206.186	1.3295
36	Pentanal	C110623	C_5_H_10_O	86.1	693.5	200.678	1.423
37	2‐Pentanone	C107879	C_5_H_10_O	86.1	683.2	195.892	1.3694
38	3‐methylbutanal	C590863	C_5_H_10_O	86.1	648.1	182.764	1.4068
39	Ethyl Acetate	C141786	C_4_H_8_O_2_	88.1	605.9	168.195	1.3348
40	2‐Butanone	C78933	C_4_H_8_O	72.1	586.8	161.951	1.2441
41	Ethanol	C64175	C_2_H_6_O	46.1	446.1	122.727	1.0477
42	Acetone	C67641	C_3_H_6_O	58.1	492.8	134.574	1.1141
43	2‐Propanol	C67630	C_3_H_8_O	60.1	503.6	137.456	1.1796

## 3 RESULTS AND DISCUSSION

### 3.1 The change of color

As shown in Figure 1, the color and form of the jujubes have great changes through blacking process. Table 2 shows the values of the color difference of the sample in the process. It could be seen that the data of 12 hr and the original date are significantly different, while the data of 12–96 hr are relatively close. It indicated that the color of the sample changes most obviously at 12 hr, after which the color of the treatment does not change much.

**Table 2:** The change of Δ*L*, Δ*a*, Δ*b*, and Δ*E* in the process of jujube blacking

时间/hr	Δ*L*	Δ*a*	Δ*b*	Δ*E*
0	−0.43	−1.95	−0.69	2.12
12	−1.69	−9.68	−2.07	10.04
24	−1.47	−9.45	−2.01	9.77
36	−1.01	−8.76	−1.87	9.02
48	−1.55	−9.69	−1.96	10.01
60	−1.70	−10.41	−2.16	10.77
72	−1.53	−10.02	−2.14	10.36
84	−0.74	−8.20	−1.85	8.44
96	−0.96	−8.84	−1.92	9.10

### 3.2 The change of total acid contents during the thermal processing of black jujube

**Figure 2:**
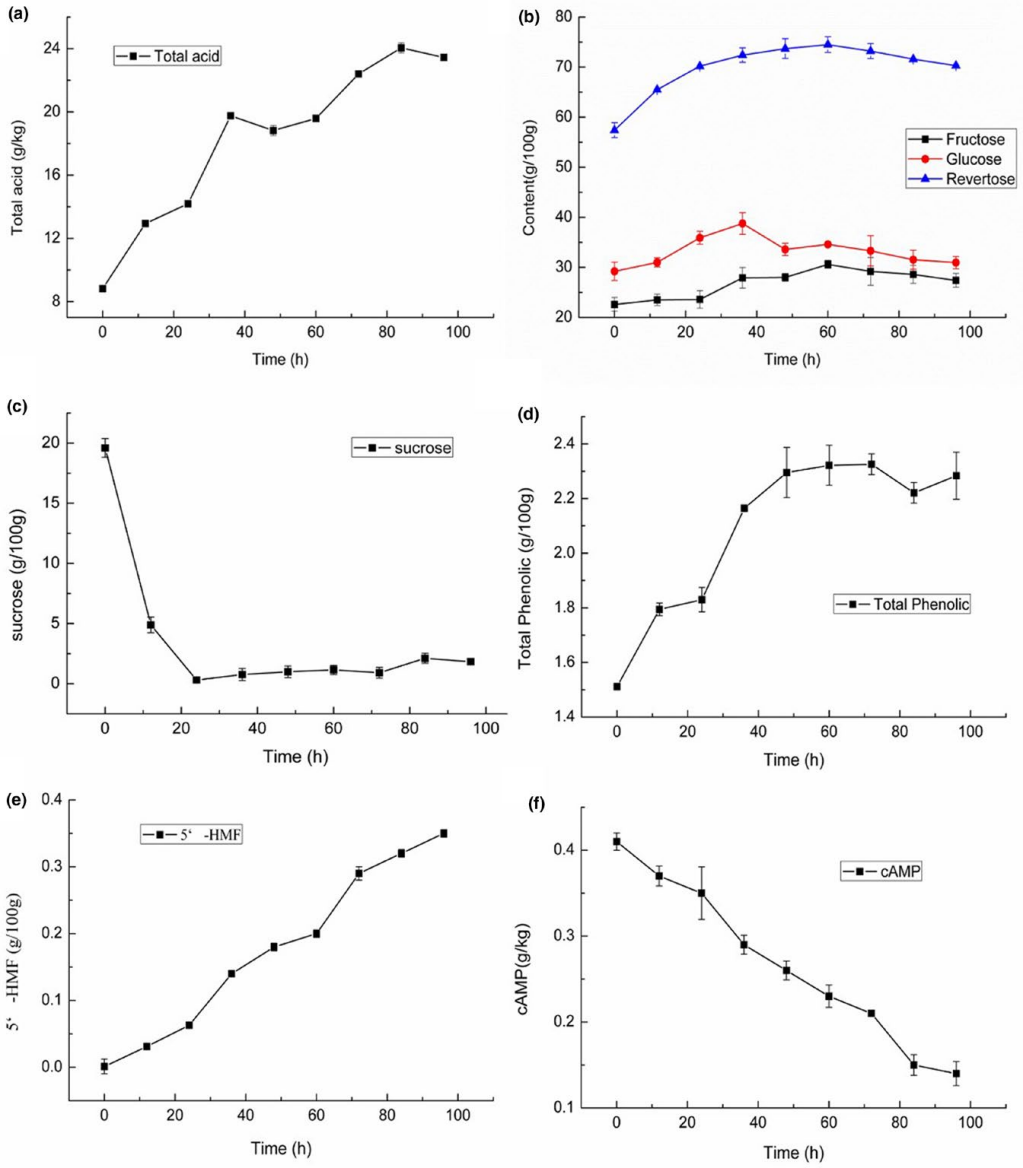
The changing tendency of total acid (a), sugar (b), sucrose (c), total phenolic (d), 5‐HMF (e), and cAMP (f) in aging process

Figure 2a presents the changes in the total acid contents during the aging period of the jujube fruits. The total acid content of dried jujube was mild with 8.82 g/kg and increased to 23.45 g/kg by 177.21% with thermal processing for 96 hr (Table 3). The rate of acid formation was different, and it was assumed to be related to nonenzymatic browning due to early color changes when the acidity increased faster. The acid increase was partially associated with the production of carboxylic acids during the browning reactions, which have been reported to be generated via the oxidation of the aldehyde in aldohexose (Sang, Cho, Yong, Lee, & Park, 2014). It also has been established that a variation in organic acids may occur during the Maillard reaction due to the co‐existence of amino and carbonyl groups.

**Table 3:** Contents (dw, mean ± *SD*, *n* = 3) of the ingredients in samples with different aging stages

Analytes (g/kg)	Aging stages (hr)								
	0	12	24	36	48	60	72	84	96
Fructose	226.3 ± 13.7	235.1 ± 11.4	236.7 ± 17.5	278.6 ± 20.8	280.3 ± 7.8	306.3 ± 7.8	291.7 ± 27.6	286.2 ± 17.9	274.1 ± 13.6
Glucose	292.4 ± 18.3	310.2 ± 8.9	359.0 ± 12.7	387.7 ± 21.7	335.8 ± 12.2	346.3 ± 0.9	332.9 ± 30.2	315.2 ± 19.3	309.5 ± 12.2
Sucrose	195.6 ± 7.8	48.9 ± 6.4	3.1 ± 0.8	7.6 ± 5.1	9.9 ± 4.9	11.5 ± 3.9	9.1 ± 1.5	21.1 ± 4.1	18.3 ± 1.9
Reducing sugar	574.3 ± 15.4	655.2 ± 1.2	701.9 ± 10.2	723.5 ± 14.4	737.2 ± 19.8	745.0 ± 15.5	731.7 ± 15.0	715.6 ± 1.1	703.0 ± 5.8
Total acid	8.82 ± 0.02	12.94 ± 0.15	14.19 ± 0.01	19.76 ± 0.01	18.82 ± 0.32	19.59 ± 0.03	22.4 ± 0.06	24.05 ± 0.32	23.05 ± 0.02
Total phenolic	15.1 ± 1.3	17.9 ± 0.2	18.3 ± 0.4	21.6 ± 0.1	23.0 ± 0.9	23.2 ± 0.7	23.3 ± 0.4	22.2 ± 0.4	22.8 ± 0.9
5′‐HMF	0.01 ± 0.11	0.29 ± 0.01	0.64 ± 0.02	1.38 ± 0.03	1.81 ± 0.06	2.05 ± 0.06	2.89 ± 0.01	3.23 ± 0.06	3.52 ± 0.06
cAMP	0.41 ± 0.01	0.37 ± 0.01	0.35 ± 0.03	0.29 ± 0.01	0.26 ± 0.01	0.23 ± 0.01	0.21 ± 0.01	0.15 ± 0.01	0.14 ± 0.02

### 3.3 Sugar content during the blackening process

The changes in sugar contents in black jujube at different aging stages are presented in Figure 2. As shown in Figure 2b, increasing trends were found for the contents of total reducing sugar, glucose, and fructose during pre‐period aging. Fructose, glucose, and total reducing sugar increased by 35.40% on 60 hr, 32.76% on 36 hr, and 29.79% on 60 hr, respectively. Otherwise, their content began to decrease during the later period of aging. The sucrose content was exactly opposite to that of the reducing sugar during aging, and it rapidly reduced from 195.6 to 3.1 g/kg in 24 hr (Table 3) and then changed slowly (Figure 2c). Because sucrose is decomposed into monosaccharides or disaccharides, which have reducibility, the results could indicate that sucrose may be converted to reducing sugar with sample aging. The relevance between the trends of sucrose and reducing sugar was analyzed, and the correlation coefficient was −0.934; the results showed a bilateral significant correlation. At the end of aging, the content of reducing sugar, which is one of the reactants of the Maillard reaction, decreased because of continued consumption. In addition, the total amount of glucose and fructose accounted for 83.0%–90.1% of the reducing sugar during the aging process. This confirmed that fructose and glucose are the main contents of the reducing sugar.

### 3.4 Content of functional components during the blackening process

Changes in the chemical functional composition, including HMF, total phenolic, and cAMP, were studied during the aging process. The contents of total polyphenols were constantly enhanced during pre‐period processing (0–4 8 hr), the amount of the compound increased with treatment by 50.99% compared to raw jujube, and it then remained basically the same (48–96 hr) (Figure 2d). An increase in these properties in black garlic compared with those in raw garlic due to heating at high temperatures has also been reported (Nencini, Menchiari, Franchi, & Micheli, 2011; Shin et al., 2008). The increasing content of polyphenol was considered to be caused by the release of these compounds to the outside or by the conversion of some other ingredients into polyphenols during the heat processing of aging jujube (Kim et al., 2013).

As shown in Figure 2e and Table 3, HMF that almost nonexistent in raw jujube rapidly increased to 3.5 g/kg by the blacking process of 96 hr. HMF mainly formed via the Maillard reaction, which is regarded as the most important contaminant that is present in heat‐induced products, especially in bakery food (Capuano & Fogliano, 2011). The results could be associated with the fact that HMF is produced at high temperature.

The raw samples had higher contents of cAMP (4.1 g/kg DW) compared to the black samples in which the trend of contents decreased as shown in Figure 2f and dramatically dropped to 1.4 g/kg DW by 65.85% (Table 1). This confirms that cAMP was consumed during nonenzymatic browning during the process.

### 3.5 Change in the aroma ingredients

**Figure 3:**
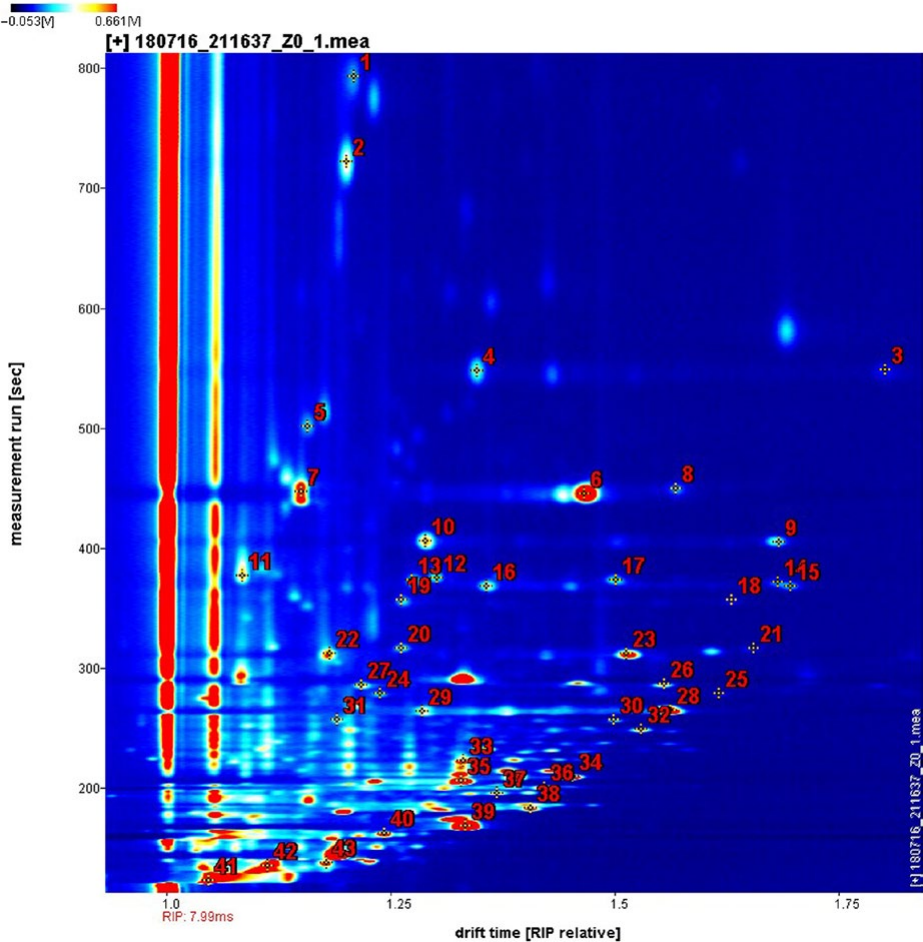
Gas phase ion mobility spectrogram of the sample

**Figure 4:**
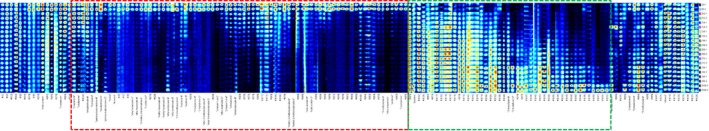
The Gallery Plot of volatile components in black jujubes

The content of volatile organic compounds between samples during 0–96 hr blackening was compared using Gallery Plot. The result automatically generated a fingerprint map in Figure 3 arranged from top to bottom according to processing time. Each row represents a sample, and each column represents a substance. As shown in Figure 4, the flavor change of jujube is obvious. The content of benzaldehyde, γ‐butyrolactone, ethyl hexanoate, 1‐octene‐3‐ol, methyl hexanoate, ethyl valerate, heptaldehyde, 2‐heptanone, ethyl 3‐methylbutanoate, 2‐hexen‐1‐ol, ethyl butyrate, butyl acetate, 3‐methylbutanol, valeraldehyde, pentanone, and other substances gradually decreased until disappearing in the process of red frame region. The material in the green frame area is slowly produced during the fermentation process and increases with time, such as acetone, 2‐acetylfuran, and some indeterminate compounds.

**Figure 5:**
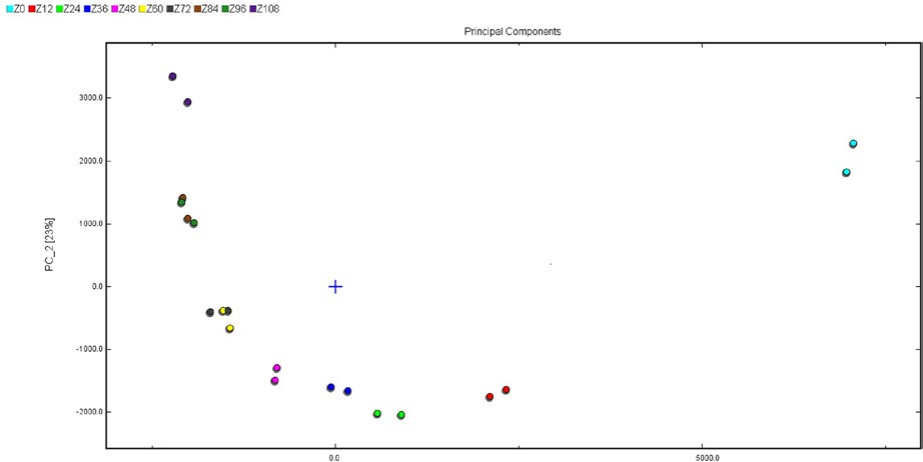
The PAC analysis of jujube samples

The result of PAC analysis on ingredients data was shown in Figure 5. The volatile components of raw jujube (Z0) were obviously different from processed jujube (Z12‐Z108). The components of Z60 and Z72 were very similar, and the same situation occurs in Z84 and Z96. It demonstrated that the volatile components change slowly in the later period, and there is a significant difference.

## 4 CONCLUSIONS

In this study, black jujube was prepared via high temperature aging from untreated jujube, and the contents of sugar, acid, total phenols acid, 5′‐HMF, cAMP, and volatile ingredients in jujube were monitored. The results revealed that black jujube is richer in reducing sugar, 5′‐HMF and phenolic, which leads to a corresponding increase in antioxidant properties, but it lacks cAMP.

It shown that both colorinternal composition and internal composition changes in the high temperature and high humidity processing environment. A quantitative analysis of the contents of reducing sugar, total phenols, and 5′‐HMF present in black jujube is considered as great importance for evaluating the effect of black jujube aging. This result is of interest to nutritionists and consumers as new jujube products will be developed to enhance the health functionality of jujubes

## ETHICAL STATEMENTS

This study does not involve any human or animal testing.

## CONFLICT OF INTEREST

The authors declare that they do not have any conflict of interest.
